# Parasitic Infection Improves Survival from Septic Peritonitis by Enhancing Mast Cell Responses to Bacteria in Mice

**DOI:** 10.1371/journal.pone.0027564

**Published:** 2011-11-16

**Authors:** Rachel E. Sutherland, Xiang Xu, Sophia S. Kim, Eric J. Seeley, George H. Caughey, Paul J. Wolters

**Affiliations:** Department of Medicine, University of California San Francisco, San Francisco, California, United States of America; University of Giessen Lung Center, Germany

## Abstract

Mammals are serially infected with a variety of microorganisms, including bacteria and parasites. Each infection reprograms the immune system's responses to re-exposure and potentially alters responses to first-time infection by different microorganisms. To examine whether infection with a metazoan parasite modulates host responses to subsequent bacterial infection, mice were infected with the hookworm-like intestinal nematode *Nippostrongylus brasiliensis,* followed in 2–4 weeks by peritoneal injection of the pathogenic bacterium *Klebsiella pneumoniae*. Survival from *Klebsiella* peritonitis two weeks after parasite infection was better in *Nippostrongylus*-infected animals than in unparasitized mice, with *Nippostrongylus*-infected mice having fewer peritoneal bacteria, more neutrophils, and higher levels of protective interleukin 6. The improved survival of *Nippostrongylus*-infected mice depends on IL-4 because the survival benefit is lost in mice lacking IL-4. Because mast cells protect mice from *Klebsiella* peritonitis, we examined responses in mast cell-deficient *Kit^W-sh^/Kit^W-sh^* mice, in which parasitosis failed to improve survival from *Klebsiella* peritonitis. However, adoptive transfer of cultured mast cells to *Kit^W-sh^/Kit^W-sh^* mice restored survival benefits of parasitosis. These results show that recent infection with *Nippostrongylus brasiliensis* protects mice from *Klebsiella* peritonitis by modulating mast cell contributions to host defense, and suggest more generally that parasitosis can yield survival advantages to a bacterially infected host.

## Introduction

Despite availability of antibiotics, more than 100,000 deaths are attributable to sepsis every year, making it the fifth leading cause of death in the United States [1]. Septic patients succumb to mediators of the innate immune response to infection [2]. These mediators recruit and activate inflammatory cells that control the infection. However, when in excess, these mediators promote sepsis-associated life-threatening pathologies, including clotting activation, hypotension, lung injury, and multi-organ failure. Conversely, a number of cells (e.g., macrophages and mast cells) and mediators (e.g., TNF-α and IL-6) improve host survival following severe bacterial infections. These cells and mediators protect the host in a variety of ways, including enhancing clearance of the infecting microorganism or limiting the extent of inflammation. The ability to mount an inflammatory response sufficient to clear infection while minimizing toxicity to host tissues is a key determinant of survival during sepsis.

Symbiosis describes the relationship between organisms of different species. When the relationship benefits both organisms it is mutualistic and when it benefits just one organism, it is parasitic. Typically, intestinal worms such as nematodes are classified as parasites because they provide no apparent benefit to the host, while causing diarrhea and anemia and consuming nutrients required for growth and reproduction [3]. However, benefits of parasites may be context-dependent and less obvious than their drawbacks. The studies in the present work explore the hypotheses that intestinal parasites, which alter host defenses by provoking strong immune responses, provide a previously unrecognized benefit by altering immune responses such that the likelihood of surviving severe bacterial infection and sepsis is enhanced.

## Materials and Methods

### Materials

All chemicals were from Sigma (St. Louis, MO) unless otherwise noted.

### Experimental animals

C57BL/6 and IL-4 ^−/−^ mice were purchased from Jackson Labs (Bar Harbor, ME). Mast cell-deficient C57BL/6 *Kit^W-sh^/Kit^W-sh^* mice (*Wsh* mice) [4] were provided originally by Peter Besmer (Memorial Sloan-Kettering Institute, New York, NY). All mice used in these experiments are in a C57BL/6 background. The experimental procedures were performed in 8–12 week-old mice and were approved by the UCSF Committee on Animal Research #AN079696-03.

### *Nippostrongylus brasiliensis* infection of mice

Using previously described methods [5], mice were infected by injecting 500 *N. brasiliensis* larvae suspended in 200 µl of PBS subcutaneously at the base of the tail of each mouse via a 27-gauge needle.

### Induction of *Klebsiella pneumoniae* septic peritonitis in mice

*Klebsiella pneumoniae bacteria* (strain 43816, serotype 2, American Type Culture Collection, Manassas, VA) were resuspended in 5 mL of Nutrient Broth (Difco) and cultured overnight at 37°C. 100 µl of this suspension was added to 50 mL of Nutrient Broth and grown for 3–4 h to log phase when colony-forming units (CFU) were determined by OD_600_ readings and confirmed by culture. Septic peritonitis was generated in mice by i.p. injection of 150 CFU suspended in 200 µl of PBS. After recovery from anesthesia, mice were monitored three times daily. Moribund mice were euthanized by CO_2_ inhalation and cervical dislocation.

### Mast cell culture from bone marrow

Mouse bone marrow-derived cultured mast cells (BMCMC) were differentiated from femoral bone marrow by culture in medium supplemented with recombinant mouse IL-3 and stem cell factor(rmSCF; Peprotech, Rocky Hill, NJ) as described [6]. Cells were used after 5 weeks in culture, at which time they consisted of >95% mast cells (as identified by metachromatic granules in cells stained with toluidine blue). BMCMC from IL-4 ^−/−^ bone marrow showed similar granular morphology, levels of active tryptase, and expression of F_c_εRIα and CD117, indicating that they mature similarly when cultured in the presence of IL-3 and SCF.

### Adoptive transfer of mast cells into *Kit^W-sh^/Kit^W-sh^* mice

To reconstitute intraperitoneal mast cells, 4×10^6^ BMCMC suspended in 500 µl of sterile PBS were injected i.p into 5-week old *Kit^W-sh^/Kit^W-sh^* mice. Reconstituted mice were used in experiments after allowing 5 weeks for mast cells to differentiate within the peritoneum [7], [8]. This method selectively reconstitutes mast cells in peritoneum and mesentery to levels similar to those in wild type C57BL/6 mice. In additional experiments, mice were rapidly reconstituted to ensure that the phenotype of mast cells studied in vivo matched the in vitro phenotype. In these experiments, 1.25×10^5^ BMCMC suspended in 500 µl of sterile PBS were injected i.p. into 5-week old *Kit^W-sh^/Kit^W-sh^* mice, which were studied within 24 h of injection.

### Quantification of cellular response to infection

To recover intraperitoneal inflammatory cells, anesthetized mice were euthanized and the abdominal skin cleansed with 70% ethanol. 4 mL of sterile 0.9% NaCl were then instilled into the peritoneum. The abdomen was massaged gently for 1 min then opened with sterile scissors to reclaim lavage fluid, which was centrifuged at 500 *g* for 4 min at 4°C and the supernatant saved for cytokine analysis. Cell pellets were resuspended in red cell lysis buffer (Sigma) for 10 min, re-centrifuged, and the cell pellet resuspended in PBS. Cell numbers were counted with a hemocytometer and cells differentiated in cytospun preparations stained with Diff-Quik (American Scientific Products, McGaw Park, IL).

### Quantification of bacterial CFU

10 µl of peritoneal lavage fluid were diluted serially in sterile 0.9% NaCl. 10 µl of each dilution were aseptically plated and cultured at 37°C on nutrient agar for non-fastidious microorganism plates. After 24 h, the numbers of bacterial colonies were counted.

### Cytokine analysis

Cytokine concentrations were measured in lung and peritoneal lavage fluid using ELISA kits: MIP-2 (R&D systems, Minneapolis, MN), KC (R&D systems), TNF-α (eBioscience, San Diego, CA), IL-6 (R&D Systems), and IFN-γ (eBioscience) according to the manufacturers' protocols.

### Statistics

Survival curves were analyzed using the log-rank (mantel-cox) test. ANOVA followed by two-tailed *t* testing was used to compare markers of organ dysfunction, bacterial CFU, and mean cytokine concentration. Calculations were performed using Statview 5.0.1 software (SAS Institute Inc., Cary, NC) and graphpad PRISM software (GraphPad, La Jolla, CA). Significance was assigned to *P* values <0.05.

## Results

### Parasitosis improves survival following subsequent bacterial infection

**Figure 1:**
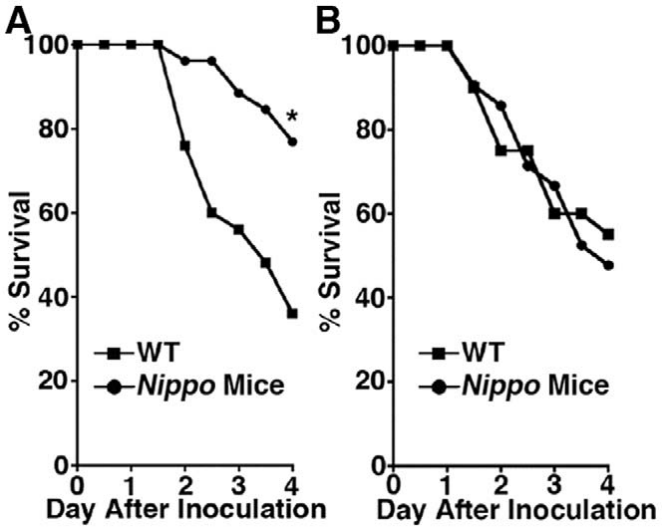
*N. brasiliensis* infection protects from death from *K. pneumoniae*-mediated septic peritonitis. C57BL/6 mice (WT) or C57BL/6 mice infected with *N. brasiliensis* (*A*) 2 or (*B*) 4 weeks earlier (*Nippo* mice) were injected with 150 CFU of *K. pneumoniae* i.p. and survival was monitored. Mice infected with *N. brasiliensis* 2 but not 4 weeks before bacterial infection are more likely to survive septic peritonitis than unparasitized control mice. (n = 25 mice/group, **P = 0.0018).

While investigating the role of mast cells in modulating survival during septic peritonitis, we noted that control mice had better survival while our mouse colony was inadvertently infected with pinworm. This observation prompted us to hypothesize that parasitosis protects against subsequent bacterial infection. To examine this hypothesis, *N. brasiliensis*-infected mice (henceforth termed *Nippo* mice) were infected i.p. with *K. pneumoniae* 2 weeks later (after worm expulsion[9]) to induce septic peritonitis. Remarkably, survival following *K. pneumoniae*-mediated septic peritonitis in *Nippo* mice was significantly better than in unparasitized control mice (**Fig. 1A**). Interestingly, the survival advantage was no longer present in mice infected with *Klebsiella* 4 weeks after *N. brasiliensis* infection (**Fig. 1 *B***), suggesting that the protective mechanism does not involve a permanent change in adaptive immunity.

### Recent parasitosis enhances peritoneal clearance of bacteria

**Figure 2:**
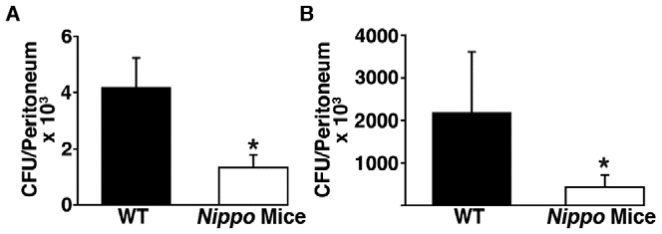
Peritoneal clearance of *K. pneumoniae* is enhanced in mice previously infected with *N. brasiliensis*. C57BL/6 (WT) and C57BL/56 mice infected 2 weeks earlier with *N. brasiliensis* (*Nippo* Mice) were injected i.p. with 150 CFU of *K. pneumoniae*. Dilutions of peritoneal lavage fluid obtained 4 (A) and 24 h (B) after *K. pneumoniae* injection were cultured on agar plates and bacterial colonies were counted. (n = 7–9 mice/group, *P<0.05).

To probe mechanisms by which *N. brasiliensis* infection protects mice from subsequent septic peritonitis, endpoints known to affect sepsis survival, including bacterial clearance, degree and type of cellular inflammation, and levels inflammatory cytokines, were assessed at intervals after infection. The results show that peritoneal CFU of *K. pneumoniae* are markedly diminished 4 and 24 h after inoculation in *Nippo* versus unparasitized mice (**Fig. 2**), suggesting that improved survival may be due to enhanced peritoneal clearance of bacteria.

### Recent parasitosis enhances peritoneal recruitment of neutrophils in response to bacteria

**Figure 3:**
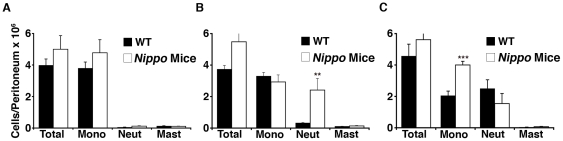
Peritoneal neutrophil recruitment is enhanced in parasitized mice following *K. pneumoniae* infection. C57BL/6 (WT) and C57BL/6 mice infected 2 weeks earlier with *N. brasiliensis* (*Nippo* mice) were euthanized at baseline (*A*), 4 h (*B*) and 24 h (*C*) after i.p. injection of 150 CFU of *K. pneumoniae* and inflammatory cells were recovered by peritoneal lavage. Total cells were counted using a hemocytometer and differential cell counts were determined on cytospun cells stained with Diff-Quik. (n = 7–9 mice/group, **P<0.01, ***P<0.001 for *Nippo* compared to unparasitized control mice).

To decipher the mechanism by which *N. brasiliensis* enhances *K. pneumoniae* clearance, peritoneal inflammatory cells were quantified in *Nippo* mice at baseline and 4 and 24 h after *K. pneumoniae* infection. At baseline, there was no difference in the number or types of peritoneal leukocytes in *Nippo* versus unparasitized mice. However, *Nippo* mice had significantly more neutrophils 4 h after inoculation with *K. pneumoniae* (**Fig. 3**). By 24 h after inoculation, this difference in neutrophils is no longer present and there is a slight predominance of monocytes in *Nippo* mice, possibly related to fewer bacteria (**Fig. 2**). These results indicate that prior infection with *N. brasiliensis* enhances local neutrophil recruitment during peritoneal *K. pneumoniae* infection and that this early recruitment likely enhances bacterial clearance and survival.

### Il-6 release increases in *Nippo* mice after *K. pneumoniae* infection

**Figure 4:**
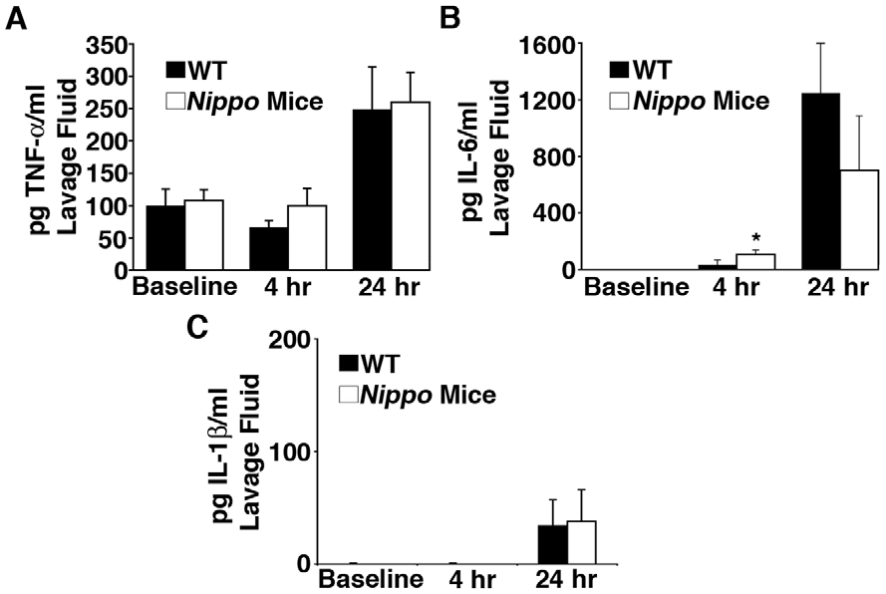
IL-6 is elevated in *Nippo* mice. Levels of (*A*) TNF-α, (*B*) IL-6, and (*C*) IL-1β were measured by ELISA in peritoneal lavage fluid from WT or *Nippo* mice at baseline, 4 and 24 h after i.p. injection of 150 CFU of *K. pneumoniae*. (n = 7–9 mice/time point, *P<0.05 for *Nippo* mice compared to unparasitized control mice).

To investigate whether differences in cytokine expression explain enhanced bacterial clearance in *Nippo* mice, intraperitoneal levels of three cytokines that regulate the host response to bacterial infections (TNF-α, IL-6, and IL-1ß) were compared at baseline and 4 and 24 h after *K. pneumoniae* infection in *Nippo* and unparasitized mice. Baseline levels of these cytokines were similar in *Nippo* and unparasitized mice (**Fig. 4**), indicating that differences in baseline production of key cytokines that mediate innate immune responses to bacterial infection do not explain the differences in bacterial clearance. Although peritoneal levels of TNF-α and IL-1ß did not differ 4 or 24 h after *K. pneumoniae* infection, levels of IL-6 at 4 h were significantly higher in *Nippo* mice (**Fig. 4**). IL-6 enhances neutrophil killing of *K. pneumoniae* in the peritoneum within the first hours of inoculation [10]. This suggests that the higher intraperitoneal levels of IL-6 early after *K. pneumoniae* infection may contribute to enhanced bacterial clearance and higher likelihood of surviving infection.

### Improved survival of *Nippo* mice is mast cell-dependent

**Figure 5:**
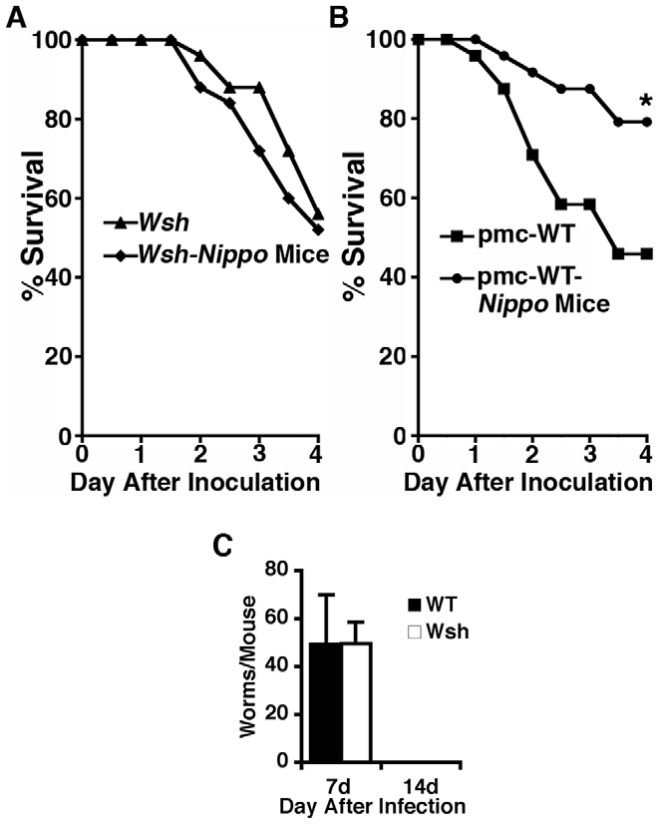
Improved survival of *Nippo* mice is mediated by mast cells. (*A*) Mast cell-deficient *Kit^W-sh^/Kit^W-sh^* mice (*Wsh*, n = 25) or *Wsh* mice infected with *N. brasiliensis* 2 weeks earlier (*Wsh+Nippo* mice, n = 24) were injected with 150 CFU of *K. pneumoniae* i.p. and survival was monitored. (*B*) pmc-WT (25 mice) and pmc-WT mice infected with *N. brasiliensis* 2 weeks earlier (pmc-WT-*Nippo* Mice, 25 mice), were injected with 150 CFU of *K. pneumoniae* i.p. and survival monitored (*P<0.012). (*C*) Expulsion of *N. brasiliensis* is normal in *Kit^W-sh^/Kit^W-sh^* mice. WT and *Kit^W-sh^/Kit^W-sh^* mice (n = 5/group) were infected with 500 *N. brasiliensis* larvae and intestinal worm burden assessed 7 and 14 d after infection.

Because two endpoints (i.e., numbers of locally recruited neutrophils and levels of intraperitoneal IL-6) that could influence survival from bacterial peritonitis are mast-cell dependent in some disease models [10], [11], [12], [13], we examined whether mast cells mediate the observed survival advantage in *Nippo* mice. To test this possibility, mast cell-deficient *Wsh* mice and *Wsh* mice infected 2 weeks earlier with *N. brasiliensis* (*Wsh-Nippo* mice) were subjected to *K. pneumoniae* septic peritonitis. In contrast to the survival advantage noted in wild type mice, there was no difference in survival between *Wsh* and *Wsh-Nippo* mice (**Fig. 5** ***A***), suggest that mast cells may be essential for the survival advantage. To determine whether this protective effect is due to mast cells specifically, survival from *K. pneumoniae* peritonitis was compared in *N. brasiliensis-*infected and unparasitized *Wsh* mice in which peritoneal mast cells were selectively reconstituted by injection of BMCMC from wild type mice [14], [15]. As shown in **Fig. 5B**, mast cell reconstitution in *Wsh* mice recovered the survival advantage conferred by prior parasitosis. Because *Wsh* mice clear *N. brasiliensis* worms normally (**Fig. 5** ***C***), worm retention does not explain the lack of a survival advantage in *Wsh-Nippo* mice. Similar to wild type *Nippo* mice, mast cell-reconstituted *Wsh-Nippo* mice had higher levels of IL-6 in peritoneal lavage fluid compared to mast cell-reconstituted *Wsh* control mice 4 h after infection with *Klebsiella* (124 vs. 65.4 pg/mL). These findings suggest that the survival advantage enjoyed by wild type *Nippo* mice is mast cell-dependant.

### IL-4 favorably alters mast cell responses to sepsis

**Figure 6:**
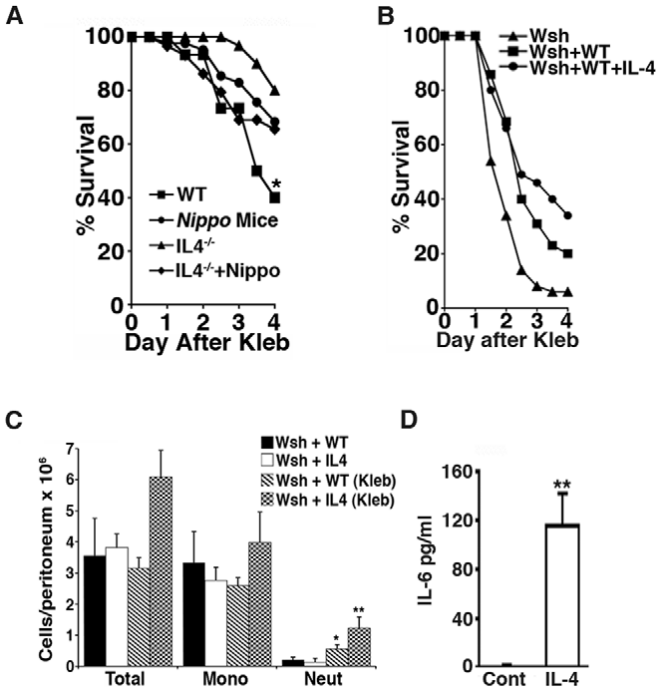
IL-4 favorably alters mast cell responses to sepsis. (*A*) IL-4 ^+/+^ mice (WT), IL-4 ^+/+^ mice infected with *N. brasiliensis* 2 weeks earlier (*Nippo* mice), IL-4 ^−/−^ mice, or IL-4 ^−/−^ mice infected with *N. brasiliensis* 2 weeks earlier (IL-4 ^−/−^ *+*Nippo) were injected with 150 CFU of *K. pneumoniae* i.p and survival was monitored. (n = 35 mice/group, *P = 0.02 comparing WT vs. *Nippo* mice and *P* = 0.13 comparing IL-4 ^−/−^ vs. IL-4 ^−/−^ *+*Nippo). (*B*) *Wsh* mice (Wsh) were reconstituted by i.p. injection of 125,000 WT BMCMC (Wsh+WT) or BMCMC preconditioned for 7 d with 50 ng/mL IL-4 (Wsh+WT+IL4). 24 h later, mice were injected with 150 CFU of *K. pneumoniae* i.p. Mice reconstituted with BMCMC conditioned with IL-4 were more likely to survive than controls (n = 35 mice/group). (*C*) IL-4 enhances mast cell-dependent neutrophil recruitment during *K. pneumoniae* septic peritonitis. Wsh+WT and Wsh+IL4 mice were injected with 150 CFU of *K. pneumoniae* i.p. Inflammatory cells in peritoneum of uninfected *Wsh*-WT (black bar) or *Wsh*-IL-4 (white bar) and infected *Wsh*-WT (*Wsh*-WT-kleb, hatched bar) and infected *Wsh*-IL-4 (*Wsh*-IL-4-kleb, boxed bar) mice 4 h after infection with *Klebsiella*. (*P<0.05 Wsh-WT vs. Wsh-WT (kleb) neut, and **P<0.01 Wsh-IL4-kleb vs. Wsh-WT-kleb neuts). (*D*) IL-4 conditioned mast cells produced greater amounts of IL-6. BMCMC cultured for 7 d in the absence (cont) or presence of 50 ng/mL IL-4 (IL-4) were stimulated with heat-killed *K. pneumoniae* and the amount of IL-6 released into the culture media quantified by ELISA. (**P<0.01).

Th2 cytokines are major mediators of the immune response to *N. brasiliensis* infection [5]. To examine whether Th2 cytokines could contribute to the survival advantage enjoyed by *Nippo* mice, we focused on IL-4, a Th2 cytokine released during *N. brasiliensis* infection that is not required for worm expulsion [16]. To test whether IL-4 contributes to the survival advantage, survival of parasitized and unparasitized wild type and IL-4 ^−/−^ mice was compared in the *Klebsiella* septic peritonitis model. In these experiments, survival of unparasitized IL-4 ^−/−^ and parasitized IL-4 ^−/−^ mice was similar to each other (**Fig. 6** ***A***), suggesting that the survival advantage in wild type *Nippo* mice is IL-4-dependent.

To test whether IL-4 alters mast cell responses to *Klebsiella* infection and sepsis, *Wsh* mice were reconstituted with BMCMC cultured for 1 week in the absence (*Wsh*+WT) or presence of 50 ng/mL IL-4 (*Wsh*+WT+IL-4). 10 week-old mice were reconstituted by i.p. injection of 125,000 mast cells, then infected i.p. 24 h later with *K. pneumoniae*. As shown in **Fig. 6B**, *Wsh* mice reconstituted with IL-4-conditioned mast cells are more likely to survive peritonitis than *Wsh* mice reconstituted with unconditioned mast cells.

To explore mechanisms by which IL-4-conditioned mast cells enhance survival from septic peritonitis, peritoneal inflammatory cell profiles in *Wsh*+WT and *Wsh*+WT+IL-4 mice were compared at baseline and 4 h after i.p. injection of *Klebsiella*. Uninfected *Wsh*+WT and *Wsh*+WT+IL-4 mice did not differ in total differential cell counts, indicating that IL-4 conditioning of BMCMC does not influence the baseline profile of peritoneal inflammatory cells (**Fig. 6C**). In contrast, *Wsh*+WT+IL-4 mice recruited significantly more neutrophils than *Wsh+*WT controls 4 h after *Klebsiella* infection, as also observed in wild type *Nippo* mice (**Fig. 3**), suggesting that IL-4 conditioning of mast cells in vivo contributes to the survival advantage in *Nippo* mice.

To examine whether IL-4 conditioning of mast cells can contribute to the observed increased levels of IL-6 in *Nippo* mice, IL-6 production was measured in BMCMC conditioned for one week with IL-4 followed by 24 h of incubation with heat-killed *K. pneumoniae*. As shown in **Fig. 6D**, IL-4-conditioned mast cells produce significantly more IL-6 than unconditioned mast cells following stimulation with *K. pneumoniae*, consistent with a role for IL-4 in priming mast cells to respond to the presence of bacteria in vivo by producing IL-6.

## Discussion

This study reports that prior infection of mice with parasitic worms can improve survival during subsequent septic peritonitis. In this model, a recent history of parasitosis with the hookworm-like nematode *N. brasiliensis* improves survival by enhancing clearance of *K. pneumoniae* bacteria infecting the peritoneum. The worms appear to accomplish this by provoking production of Th2 cytokines, such as IL-4, which primes mast cells to enhance their innate responses to bacteria. These suggest that intestinal worm infections may be mutually beneficial to the host by protecting them from subsequent bacterial infection.

The immune system is in a constant state of flux controlled in large part by the infectious organisms it encounters. These exposures educate the immune system such that it will clear repeat infections by the same microorganism and thereby limit potential harm to the host. More recent data has shown that an infection by one microorganism can provide heterologous protection from an unrelated organism [17], [18], [19], [20]. For example, latent herpesvirus infection improves survival from subsequent *Listeria monocytogenes* infection by increasing *Listeria* clearance [17]. Similarly, inhalation of *Haemophilus influenzae* extracts improves survival from subsequent infections, including *Streptococcus pneumoniae*, *Klebsiella pneumoniae*, *Aspergillus fumigatus*, and influenza A [18], [19], [20]. Collectively, these studies show that prior exposure to infectious agents or extracts of them can favorably modulate the immune response to subsequent heterologous infections.

The present study reports parasitic infection with *N. brasiliensis* favorably alter the immune response to a heterologous intraperitoneal infection with the bacterium *K. pneumoniae*. The data show that mice previously infected with *N. brasiliensis* have more neutrophil recruitment and higher intraperitoneal IL-6 levels early after *K. pneumoniae* infection. These exaggerated immune responses are associated with improved bacterial clearance and host survival [10]. Although an explanation for the enhanced neutrophil recruitment remains undefined, possibilities include activation of innate immune cells, such as mast cells, by products made by *N. brasiliensis* or by mediators produced in response to the *N. brasiliensis* infection. This study reports IL-4 as a candidate mediator produced in response to the *N. brasiliensis* infection that can augment mast cell responses to subsequent *K. pneumoniae* infection.

Prior work has established that mast cells play an essential role in regulating the immune response to severe bacterial infections and sepsis [7], [11] by secreting of TNF-α and tryptase, which promote recruitment of neutrophils to sites of infection [21] [13], and IL-6, which potentiates neutrophil killing of bacteria [10], [12]. The finding that *Nippo* mice have higher intraperitoneal IL-6 levels 4 h after infection and that IL-4-conditioned mast cells generate more IL-6 is consistent with the idea that mast cells are a source of the higher IL-6 levels in *Nippo* mice. Determining whether mast cells are a source of IL-6 under these conditions, or other cells contribute will require further study. In prior work, we reported that increased intraperitoneal levels of IL-6 improve survival of septic DPPI ^−/−^ mice [12]. The finding that *Nippo* mice have higher intraperitoneal IL-6 levels underscores the beneficial effects of locally produced IL-6 and suggests that increasing peritoneal IL-6 early in the course of infection benefits the host independent of its cellular source.

The observed mast cell dependence of the survival advantage of parasitized mice with bacterial peritonitis suggests that *N. brasiliensis* infection changes mast cell behavior and that mast cell responses to bacterial infection are not static. This is consistent with reports that mast cell numbers and phenotypes change during parasitic infections [5], [22], [23]. In addition, this study provides the first evidence that the phenotypic change produced by *N. brasiliensis* infection in vivo or by conditioning with IL-4 in vitro protects mice from death from *K. pneumoniae*-mediated septic peritonitis. These findings suggest that augmenting mast cell responses to bacteria could be therapeutic in severe bacterial infections and sepsis.

Although not required for worm expulsion, IL-4 is a major mediator of the host response to *N. brasiliensis* infection [24]. The improved survival of *Nippo* mice appears to depend on IL-4 because the survival benefit is lost in mice lacking this cytokine (**Fig. 6**). Although IL-4-conditioned BMCMC are not phenocopies of peritoneal MC in *Nippo* mice, the result that mast cell-deficient *Wsh* mice reconstituted with IL-4-conditioned mast cells have better survival and elevated intraperitoneal IL-6 levels following *K. pneumoniae* peritonitis suggests that conditioning of peritoneal mast cells by endogenously generated IL-4 in *Nippo* mice could contribute to the survival benefit. The biochemical explanation for how IL-4 increases mast cell IL-6 production remains undefined. Because the heat-killed *Klebsiella* was used as a stimulus, we suspect IL-4 is influencing there TLR signaling. Overall these findings are consistent with prior observations that over-expression of IL-4 improves survival in mice with sepsis and *Pseudomonas* pneumonia [25], [26]. Similarly, IL-4 stimulates production of the antimicrobial peptide LL37 in mast cells [27], showing that IL-4 can provoke a change in mast cells that could enhance bacterial clearance. Determining how IL-4 enhances mast cell responses in *Nippo* mice and whether other Th2 cytokines similarly contribute to the protection requires further study.

Classically, parasite-host interactions are recognized to benefit the parasite and harm the host. This perspective has changed as it has become apparent that some parasites may provide a benefit to their host [28]. For example, trypanosomes synthesize vitamin B6 and therefore can provide a source of the vitamin to infected rodents living in environments low in vitamin B6 [29]. The present study reports a novel way in which metazoan parasites may benefit their host: namely, by protecting them from a subsequent life-threatening bacterial infection. Because a live rodent is required for the life cycle of *N. brasiliensis*, the survival benefit may mutually benefit the parasite by ensuring that the host survives and is available for the parasite to complete its life cycle. Thus, in the context of severe bacterial infection, *N. brasiliensis* infection is mutually beneficial rather than parasitic. Whether similar mutually beneficial parasite-host interactions occur in infected humans remains to be examined.

In summary, the data presented here establish that prior infection with a hookworm-like intestinal parasite can improve survival from a severe Gram-negative bacterial infection. The survival benefit depends on mast cells and IL-4 and is associated with augmented neutrophil recruitment, secretion of IL-6 and accelerated bacterial clearance. The results suggest that host infection with metazoan parasites can be mutually beneficial and may reinforce co-evolution of parasite and host by protecting the host from death from bacterial infection.
